# 3-Amino-4-[4-(dimethyl­amino)­phen­yl]-4,5-dihydro-1,2,5-thia­diazole 1,1-dioxide

**DOI:** 10.1107/S1600536811021520

**Published:** 2011-06-18

**Authors:** N. Burcu Arslan, Aliye Gediz Ertürk, Canan Kazak, Yunus Bekdemir

**Affiliations:** a Department of Physics, Arts and Sciences Faculty, Ondokuz Mayıs University, TR-55139 Samsun, Turkey; b Department of Chemistry, Arts and Sciences Faculty, Ordu University, TR-52200 Ordu, Turkey; c Department of Chemistry, Arts and Sciences Faculty, Ondokuz Mayıs University, TR-55139 Samsun, Turkey

## Abstract

The title compound, C_10_H_14_N_4_O_2_S, exists in the amine tautomeric form. The dihedral angle between the benzene and thia­diazo­lidine rings is 66.54 (19)°. In the crystal, mol­ecules are linked by N—H⋯O and N—H⋯N hydrogen bonds into a layer parallel to the *ac* plane. The layers are further linked by C—H⋯O hydrogen bonds.

## Related literature

For background to and applications of sulfamides, see: Autrieth *et al.* (1940 ▶); Bermudez *et al.* (1997 ▶); Forster *et al.* (1971 ▶); Gazieva *et al.* (2000 ▶); Lawson & Tinkler (1970 ▶); Spillane & Benson (1980 ▶). For related structures; see: Gazieva *et al.* (2000 ▶); Lee *et al.* (1989 ▶).
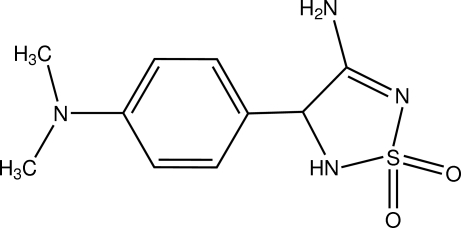


## Experimental

### 

#### Crystal data

C_10_H_14_N_4_O_2_S
*M* *_r_* = 254.32
Monoclinic, 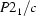

*a* = 7.2587 (8) Å
*b* = 9.8187 (8) Å
*c* = 16.893 (2) Å
β = 101.325 (10)°
*V* = 1180.6 (2) Å^3^
*Z* = 4
Mo *K*α radiation
μ = 0.27 mm^−1^
*T* = 293 K
0.44 × 0.32 × 0.21 mm

#### Data collection

Stoe IPDS 2 diffractometer
Absorption correction: integration (*X-RED32*; Stoe & Cie, 2002 ▶) *T*_min_ = 0.955, *T*_max_ = 0.985
18500 measured reflections
2615 independent reflections
2294 reflections with *I* > 2σ(*I*)
*R*_int_ = 0.042

#### Refinement

*R*[*F* ^2^ > 2σ(*F* ^2^)] = 0.038
*wR*(*F* ^2^) = 0.109
*S* = 1.06
2615 reflections
166 parameters
H atoms treated by a mixture of independent and constrained refinement
Δρ_max_ = 0.40 e Å^−3^
Δρ_min_ = −0.47 e Å^−3^

### 

Data collection: *X-AREA* (Stoe & Cie, 2002 ▶); cell refinement: *X-AREA*; data reduction: *X-RED32* (Stoe & Cie, 2002 ▶); program(s) used to solve structure: *SHELXS97* (Sheldrick, 2008 ▶); program(s) used to refine structure: *SHELXL97* (Sheldrick, 2008 ▶); molecular graphics: *ORTEP-3 for Windows* (Farrugia, 1997 ▶); software used to prepare material for publication: *WinGX* (Farrugia, 1999 ▶) and *PLATON* (Spek, 2009 ▶).

## Supplementary Material

Crystal structure: contains datablock(s) I, global. DOI: 10.1107/S1600536811021520/is2719sup1.cif

Structure factors: contains datablock(s) I. DOI: 10.1107/S1600536811021520/is2719Isup2.hkl

Supplementary material file. DOI: 10.1107/S1600536811021520/is2719Isup3.cml

Additional supplementary materials:  crystallographic information; 3D view; checkCIF report

## Figures and Tables

**Table 1:** Hydrogen-bond geometry (Å, °)

*D*—H⋯*A*	*D*—H	H⋯*A*	*D*⋯*A*	*D*—H⋯*A*
N3—H3*A*⋯O1^i^	0.87 (2)	2.16 (2)	2.947 (2)	151.0 (19)
N3—H3*B*⋯N4^ii^	0.88 (2)	2.09 (2)	2.930 (2)	160.6 (19)
C2—H2*A*⋯O2^iii^	0.959 (18)	2.416 (18)	3.038 (2)	122.3 (13)

Symmetry codes: (i) 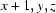; (ii) 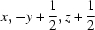; (iii) 
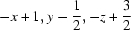
.

**Fig. 1.**
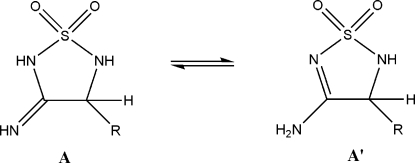
The tautomeric forms of 3-imino-1,2,5-thiadiazolidine 1,1-dioxides.

**Fig. 2.**
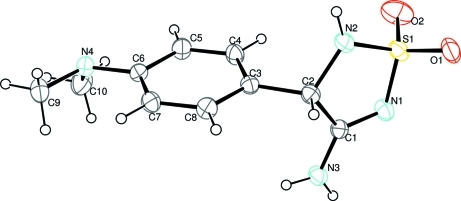
An ORTEP view of the title compound, with the atom-numbering scheme and 30% probability of displacement ellipsoids.

**Fig. 3.**
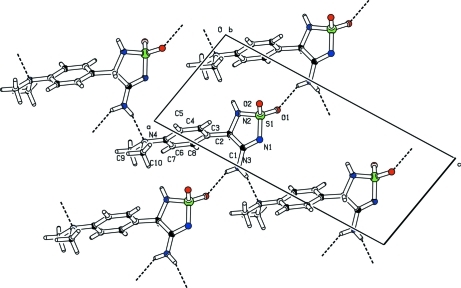
A packing diagram of the title compound, viewed down the a axis.

## supplementary crystallographic information

### Comment

Sulfamides (or sulfonamides) are well known compounds because of belonging to their pharmaceutical applications, especially in the field of antimicrobial chemotherapy. Sulfamides first used in the dye industry, but then come into question as a therapeutic antibacterial drugs for the treatment of infectious diseases. Especially were used for this purpose *p*-aminophenylsulfonamides (Bermudez *et al.*, 1997; Gazieva *et al.*, 2000). In addition, some studies revealed especially psychiatric effects of cyclic derivatives of sulfamides. They have been as well as use of insecticides and also soothing, relieving depression, pain killers and muscle relaxants (Spillane & Benson, 1980). Apart from being pharmacologically active compounds sulfamides have also been used in the making water-resistant resins (Autrieth *et al.*, 1940; Forster *et al.*, 1971; Lawson & Tinkler, 1970).

Tautomeric forms of 3-imino-1,2,5-thiadiazolidine 1,1-dioxides were studied (Gazieva *et al.*, 2000; Lee *et al.*. 1989). In solution they ocur as equilibrium mixtures of tautomers A and A^'^ (Fig. 1). This study verifies the preference of the enamine-imine tautomeric form in the solid state (Fig. 2). In the title compound all bond lenghts are in normal ranges. The benzene and the thiadiazolidine rings are planar with maximum deviations of 0.018 and 0.038 Å at atoms C6 and N2, respectively. S—O bond lenghts are 1.421 and 1.434 Å. The molecules are linked by intermolecular N—H···O, N—H···N and C—H···O hydrogen bonds (Fig. 3).

### Experimental

At 25 ml flask, 35 mg (0.72 mmol) sodium cyanide was added into 70% aqueous ethanol solution (1.3 ml) containing 70 mg (0.66 mmol) *p*-*N*,*N*-dimethyl benzaldehyde and 125 mg (1.3 mmol) sulfamide (Lee *et al.*, 1989). Reaction mixtures was heated in microwave oven at 90 W for 3 minutes. 1 N NaOH solution (0.6 ml) was added into the resulting mixture. The aqueous solution was extracted with ethyl acetate (2 × 1.3 ml) and 0.7 ml diethyl ether. The aqueous phase was then acidified with 1 N HCl (pH ~2). The green-colored solids were recrystallized in ethyl alcohol (yield 47%, m.p. 208–210 °C).

### Refinement

Atoms H2A, H3A and H3B were freely refined. Other H atoms were placed in calculated positions (C—H = 0.93 or 0.96 Å and N—H = 0.86 Å) and were included in the refinement in the riding model approximation, with *U*_iso_(H) = 1.2 or 1.5*U*_eq_(C or N).

### Crystal data

**Table:**

C_10_H_14_N_4_O_2_S	*F*(000) = 536
*M**_r_* = 254.32	*D*_x_ = 1.431 Mg m^−^^3^
Monoclinic, *P*2_1_/*c*	Mo *K*α radiation, λ = 0.71073 Å
Hall symbol: -P 2ybc	Cell parameters from 24497 reflections
*a* = 7.2587 (8) Å	θ = 2.1–27.7°
*b* = 9.8187 (8) Å	µ = 0.27 mm^−^^1^
*c* = 16.893 (2) Å	*T* = 293 K
β = 101.325 (10)°	Prism, light green
*V* = 1180.6 (2) Å^3^	0.44 × 0.32 × 0.21 mm
*Z* = 4	

### Data collection

**Table:**

Stoe IPDS 2 diffractometer	2615 independent reflections
Radiation source: sealed X-ray tube, 12 x 0.4 mm long-fine focus	2294 reflections with *I* > 2σ(*I*)
plane graphite	*R*_int_ = 0.042
Detector resolution: 6.67 pixels mm^-1^	θ_max_ = 27.2°, θ_min_ = 2.4°
ω scans	*h* = −9→9
Absorption correction: integration (*X-RED32*; Stoe & Cie, 2002)	*k* = −12→12
*T*_min_ = 0.955, *T*_max_ = 0.985	*l* = −21→21
18500 measured reflections	

### Refinement

**Table:**

Refinement on *F*^2^	Primary atom site location: structure-invariant direct methods
Least-squares matrix: full	Secondary atom site location: difference Fourier map
*R*[*F*^2^ > 2σ(*F*^2^)] = 0.038	Hydrogen site location: inferred from neighbouring sites
*wR*(*F*^2^) = 0.109	H atoms treated by a mixture of independent and constrained refinement
*S* = 1.06	*w* = 1/[σ^2^(*F*_o_^2^) + (0.0564*P*)^2^ + 0.4122*P*] where *P* = (*F*_o_^2^ + 2*F*_c_^2^)/3
2615 reflections	(Δ/σ)_max_ = 0.008
166 parameters	Δρ_max_ = 0.40 e Å^−^^3^
0 restraints	Δρ_min_ = −0.47 e Å^−^^3^

### Special details

**Table:**

Geometry. All e.s.d.'s (except the e.s.d. in the dihedral angle between two l.s. planes) are estimated using the full covariance matrix. The cell e.s.d.'s are taken into account individually in the estimation of e.s.d.'s in distances, angles and torsion angles; correlations between e.s.d.'s in cell parameters are only used when they are defined by crystal symmetry. An approximate (isotropic) treatment of cell e.s.d.'s is used for estimating e.s.d.'s involving l.s. planes.
Refinement. Refinement of *F*^2^ against ALL reflections. The weighted *R*-factor *wR* and goodness of fit *S* are based on *F*^2^, conventional *R*-factors *R* are based on *F*, with *F* set to zero for negative *F*^2^. The threshold expression of *F*^2^ > σ(*F*^2^) is used only for calculating *R*-factors(gt) *etc*. and is not relevant to the choice of reflections for refinement. *R*-factors based on *F*^2^ are statistically about twice as large as those based on *F*, and *R*- factors based on ALL data will be even larger.

### Fractional atomic coordinates and isotropic or equivalent isotropic displacement parameters (Å^2^)

**Table:**

	*x*	*y*	*z*	*U*_iso_*/*U*_eq_	
C1	0.8325 (2)	0.14751 (15)	0.83505 (9)	0.0338 (3)	
C2	0.7810 (2)	0.06451 (16)	0.75795 (9)	0.0349 (3)	
C3	0.8875 (2)	0.10603 (16)	0.69385 (9)	0.0342 (3)	
C4	0.8616 (2)	0.23280 (17)	0.65646 (10)	0.0409 (4)	
H4	0.7726	0.2926	0.6694	0.049*	
C5	0.9665 (2)	0.27045 (18)	0.60042 (10)	0.0436 (4)	
H5	0.9465	0.3552	0.5757	0.052*	
C6	1.1026 (2)	0.18340 (17)	0.57999 (9)	0.0360 (3)	
C7	1.1227 (2)	0.05506 (17)	0.61548 (10)	0.0410 (4)	
H7	1.2082	−0.0064	0.6014	0.049*	
C8	1.0168 (2)	0.01820 (17)	0.67143 (10)	0.0401 (4)	
H8	1.0328	−0.0679	0.6946	0.048*	
C9	1.3411 (3)	0.1279 (3)	0.50170 (13)	0.0593 (5)	
H9A	1.4068	0.1674	0.4633	0.089*	
H9B	1.2723	0.0494	0.4783	0.089*	
H9C	1.4297	0.1013	0.5491	0.089*	
C10	1.3040 (3)	0.3598 (2)	0.54390 (13)	0.0623 (6)	
H10A	1.3742	0.3836	0.5033	0.093*	
H10B	1.3876	0.3534	0.5954	0.093*	
H10C	1.2112	0.4286	0.5462	0.093*	
N1	0.69481 (19)	0.20064 (15)	0.86387 (9)	0.0431 (3)	
N2	0.57725 (19)	0.08209 (17)	0.73433 (9)	0.0458 (4)	
H2	0.5095	0.0530	0.6900	0.055*	
N3	1.0099 (2)	0.16048 (16)	0.86985 (9)	0.0421 (3)	
N4	1.21057 (19)	0.22794 (16)	0.52365 (8)	0.0420 (3)	
O1	0.38231 (17)	0.07726 (15)	0.84236 (9)	0.0557 (4)	
O2	0.4074 (2)	0.28818 (16)	0.77361 (12)	0.0796 (5)	
S1	0.49704 (6)	0.16529 (4)	0.80460 (3)	0.04255 (15)	
H2A	0.810 (2)	−0.0288 (19)	0.7719 (10)	0.034 (4)*	
H3A	1.094 (3)	0.128 (2)	0.8452 (13)	0.057 (6)*	
H3B	1.046 (3)	0.206 (2)	0.9150 (13)	0.047 (5)*	

### Atomic displacement parameters (Å^2^)

**Table:**

	*U*^11^	*U*^22^	*U*^33^	*U*^12^	*U*^13^	*U*^23^
C1	0.0345 (7)	0.0316 (7)	0.0359 (7)	−0.0033 (6)	0.0085 (6)	0.0016 (6)
C2	0.0330 (7)	0.0332 (7)	0.0384 (7)	−0.0033 (6)	0.0069 (6)	−0.0003 (6)
C3	0.0329 (7)	0.0347 (8)	0.0348 (7)	−0.0023 (6)	0.0062 (6)	−0.0034 (6)
C4	0.0415 (8)	0.0378 (8)	0.0462 (9)	0.0087 (7)	0.0152 (7)	0.0021 (7)
C5	0.0494 (9)	0.0375 (8)	0.0463 (9)	0.0075 (7)	0.0154 (7)	0.0076 (7)
C6	0.0341 (7)	0.0422 (8)	0.0313 (7)	−0.0004 (6)	0.0056 (6)	−0.0021 (6)
C7	0.0411 (8)	0.0408 (9)	0.0425 (8)	0.0083 (7)	0.0118 (7)	−0.0026 (7)
C8	0.0469 (9)	0.0316 (7)	0.0425 (8)	0.0043 (6)	0.0105 (7)	−0.0002 (6)
C9	0.0528 (11)	0.0781 (14)	0.0528 (10)	0.0145 (10)	0.0248 (9)	0.0044 (10)
C10	0.0648 (12)	0.0711 (14)	0.0544 (11)	−0.0258 (11)	0.0199 (10)	−0.0071 (10)
N1	0.0368 (7)	0.0461 (8)	0.0481 (8)	−0.0002 (6)	0.0125 (6)	−0.0084 (6)
N2	0.0305 (7)	0.0643 (10)	0.0417 (7)	−0.0074 (6)	0.0053 (6)	−0.0045 (7)
N3	0.0324 (7)	0.0532 (9)	0.0405 (7)	−0.0038 (6)	0.0066 (6)	−0.0087 (6)
N4	0.0400 (7)	0.0512 (8)	0.0369 (7)	−0.0002 (6)	0.0126 (6)	−0.0002 (6)
O1	0.0367 (6)	0.0680 (9)	0.0675 (8)	−0.0022 (6)	0.0228 (6)	0.0048 (7)
O2	0.0696 (10)	0.0509 (9)	0.1102 (14)	0.0219 (8)	−0.0019 (9)	0.0156 (9)
S1	0.0325 (2)	0.0407 (2)	0.0549 (3)	0.00558 (15)	0.00952 (17)	0.00524 (18)

### Geometric parameters (Å, °)

**Table:**

C1—N1	1.304 (2)	C8—H8	0.9300
C1—N3	1.313 (2)	C9—N4	1.462 (2)
C1—C2	1.520 (2)	C9—H9A	0.9600
C2—N2	1.465 (2)	C9—H9B	0.9600
C2—C3	1.505 (2)	C9—H9C	0.9600
C2—H2A	0.959 (18)	C10—N4	1.471 (3)
C3—C8	1.381 (2)	C10—H10A	0.9600
C3—C4	1.392 (2)	C10—H10B	0.9600
C4—C5	1.377 (2)	C10—H10C	0.9600
C4—H4	0.9300	N1—S1	1.6184 (15)
C5—C6	1.400 (2)	N2—S1	1.6393 (15)
C5—H5	0.9300	N2—H2	0.8600
C6—C7	1.391 (2)	N3—H3A	0.87 (2)
C6—N4	1.415 (2)	N3—H3B	0.88 (2)
C7—C8	1.379 (2)	O1—S1	1.4339 (13)
C7—H7	0.9300	O2—S1	1.4210 (15)
			
N1—C1—N3	123.42 (15)	N4—C9—H9B	109.5
N1—C1—C2	117.21 (14)	H9A—C9—H9B	109.5
N3—C1—C2	119.36 (14)	N4—C9—H9C	109.5
N2—C2—C3	113.96 (13)	H9A—C9—H9C	109.5
N2—C2—C1	103.62 (13)	H9B—C9—H9C	109.5
C3—C2—C1	113.38 (13)	N4—C10—H10A	109.5
N2—C2—H2A	109.9 (10)	N4—C10—H10B	109.5
C3—C2—H2A	108.5 (10)	H10A—C10—H10B	109.5
C1—C2—H2A	107.3 (10)	N4—C10—H10C	109.5
C8—C3—C4	118.22 (15)	H10A—C10—H10C	109.5
C8—C3—C2	120.05 (14)	H10B—C10—H10C	109.5
C4—C3—C2	121.72 (14)	C1—N1—S1	109.59 (12)
C5—C4—C3	120.54 (15)	C2—N2—S1	110.17 (11)
C5—C4—H4	119.7	C2—N2—H2	124.9
C3—C4—H4	119.7	S1—N2—H2	124.9
C4—C5—C6	121.20 (15)	C1—N3—H3A	118.3 (15)
C4—C5—H5	119.4	C1—N3—H3B	122.7 (13)
C6—C5—H5	119.4	H3A—N3—H3B	119 (2)
C7—C6—C5	117.77 (15)	C6—N4—C9	115.77 (15)
C7—C6—N4	123.02 (15)	C6—N4—C10	113.99 (14)
C5—C6—N4	119.19 (15)	C9—N4—C10	110.99 (16)
C8—C7—C6	120.55 (15)	O2—S1—O1	114.33 (10)
C8—C7—H7	119.7	O2—S1—N1	109.39 (10)
C6—C7—H7	119.7	O1—S1—N1	112.09 (8)
C7—C8—C3	121.63 (15)	O2—S1—N2	111.00 (10)
C7—C8—H8	119.2	O1—S1—N2	109.98 (9)
C3—C8—H8	119.2	N1—S1—N2	99.02 (7)
N4—C9—H9A	109.5		
			
N1—C1—C2—N2	5.40 (19)	C4—C3—C8—C7	2.0 (2)
N3—C1—C2—N2	−175.73 (14)	C2—C3—C8—C7	−177.17 (15)
N1—C1—C2—C3	129.45 (15)	N3—C1—N1—S1	179.30 (13)
N3—C1—C2—C3	−51.7 (2)	C2—C1—N1—S1	−1.88 (18)
N2—C2—C3—C8	−129.18 (16)	C3—C2—N2—S1	−130.03 (12)
C1—C2—C3—C8	112.61 (16)	C1—C2—N2—S1	−6.35 (15)
N2—C2—C3—C4	51.7 (2)	C7—C6—N4—C9	2.6 (2)
C1—C2—C3—C4	−66.6 (2)	C5—C6—N4—C9	−176.21 (16)
C8—C3—C4—C5	−1.9 (2)	C7—C6—N4—C10	−127.99 (19)
C2—C3—C4—C5	177.27 (15)	C5—C6—N4—C10	53.2 (2)
C3—C4—C5—C6	−0.5 (3)	C1—N1—S1—O2	−118.20 (14)
C4—C5—C6—C7	2.8 (2)	C1—N1—S1—O1	113.90 (13)
C4—C5—C6—N4	−178.32 (16)	C1—N1—S1—N2	−2.06 (14)
C5—C6—C7—C8	−2.7 (2)	C2—N2—S1—O2	120.26 (13)
N4—C6—C7—C8	178.48 (15)	C2—N2—S1—O1	−112.22 (12)
C6—C7—C8—C3	0.3 (3)	C2—N2—S1—N1	5.36 (13)

### Hydrogen-bond geometry (Å, °)

**Table:**

*D*—H···*A*	*D*—H	H···*A*	*D*···*A*	*D*—H···*A*
N3—H3A···O1^i^	0.87 (2)	2.16 (2)	2.947 (2)	151.0 (19)
N3—H3B···N4^ii^	0.88 (2)	2.09 (2)	2.930 (2)	160.6 (19)
C2—H2A···O2^iii^	0.959 (18)	2.416 (18)	3.038 (2)	122.3 (13)

Symmetry codes: (i) *x*+1, *y*, *z*; (ii) *x*, −*y*+1/2, *z*+1/2; (iii) −*x*+1, *y*−1/2, −*z*+3/2.
